# A COMPARISON OF THE IMPACT OF ADVERSE CHILDHOOD EXPERIENCES ON BRAIN HEALTH IN LATINA AND NON-LATINA WOMEN

**DOI:** 10.1093/geroni/igad104.0358

**Published:** 2023-12-21

**Authors:** Cindy Tsotsoros

**Affiliations:** University of Rhode Island, Kingston, Rhode Island, United States

## Abstract

Early-life experiences and social determinants of health influence late-life cognitive performance, cognitive aging trajectories, and risk of Alzheimer’s disease and related dementias (ADRD). Research shows adversity during childhood plays a central role in health outcomes later in life, such that more adverse childhood experiences (ACEs) predict higher likelihood of negative physical and mental health outcomes. As ACEs may also predict neurocognitive performance and historically marginalized groups are disproportionately exposed to ACEs, this study aims to enhance diversity in research on ACEs and cognitive aging by clarifying the role of ethnicity. Latinos are 51% more likely to experience ACEs and 1.5x more likely to experience ADRD than their non-Latino counterparts. Data were collected in two studies assessing cognitive functioning in women aged 65-85 who reported no ACEs or 3+ ACEs (54 non-Latinas, Oklahoma; 50 Latinas, Rhode Island). ACEs were measured with the Adverse Childhood Experiences Questionnaire. Cognition was measured using NIH Toolbox Cognition-Battery and Automated Neuropsychological Assessment Metrics. In the non-Latina white sample (Mage=73.2), those with 3+ ACEs exhibited inferior scores on Stroop Interference, p=.050, d=-.44. The ACEs group demonstrated higher scores on List Sorting, p=.003, d=-.78. Small effects were found in Stroop Color, Stroop Color-Word, Cognition Fluid Composite, Flanker, and Cart Sort (d=-.25, -.39, -.29, .24, -.25, respectively). These findings are compared to those within the Latina sample, demonstrating the need to extend research on life course risk factors for ADRD to this underrepresented group, which is at elevated risk of both ACEs and ADRD.

